# Heme Oxygenase-1 Promotes Delayed Wound Healing in Diabetic Rats

**DOI:** 10.1155/2016/9726503

**Published:** 2015-12-20

**Authors:** Qing-Ying Chen, Guo-Guang Wang, Wei Li, Yu-Xin Jiang, Xiao-Hua Lu, Ping-Ping Zhou

**Affiliations:** ^1^Medical Department, General Hospital of Jinan Military Command, 25 Shifan Road, Jinan, Shandong 250031, China; ^2^Department of Pathophysiology, Wannan Medical College, Wuhu 241002, China; ^3^Department of Physiology, Wannan Medical College, Wuhu 241002, China

## Abstract

Diabetic ulcers are one of the most serious and costly chronic complications for diabetic patients. Hyperglycemia-induced oxidative stress may play an important role in diabetes and its complications. The aim of the study was to explore the effect of heme oxygenase-1 on wound closure in diabetic rats. Diabetic wound model was prepared by making an incision with full thickness in STZ-induced diabetic rats. Wounds from diabetic rats were treated with 10% hemin ointment for 21 days. Increase of HO-1 protein expression enhanced anti-inflammation and antioxidant in diabetic rats. Furthermore, HO-1 increased the levels of VEGF and ICAM-1 and expressions of CBS and CSE protein. In summary, HO-1 promoted the wound closure by augmenting anti-inflammation, antioxidant, and angiogenesis in diabetic rats.

## 1. Introduction

Patients with diabetes customarily suffer from chronic nonhealing ulcers, which is one of the most serious and costly complications of diabetes [1]. It is estimated that over fifteen percent of diabetic patients will develop lower extremity ulcer, which is more likely to lead to amputation. Amputation is a major cause of high mortality in diabetic patients [2, 3]. The pathogenesis of chronic diabetic ulcers is complex and still far from being fully understood; thus methods of effective treatment are lacking. However, it is widely appreciated that peripheral vasculopathy and neuropathy increased infection of diabetic wounds and impaired wound healing process [4]. Oxidative stress resulting from hyperglycemia can injure vessels by producing excessive reactive oxygen species (ROS) and cause vasculopathy known as diabetic vascular complications [5–7]. Impaired vessels lead to peripheral microcirculation and coagulation failure, which aggravate the development of diabetic ulcer [8–10]. Therefore, it may be beneficial to chronic wound healing by increasing antioxidant and angiogenesis/vasculogenesis, which alleviates impairment of vessels and improves microcirculation.

Heme oxygenase-1, an inducible enzyme, degrades heme into biliverdin, carbon monoxide (CO), and ferrous iron. HO-1 protein expression can be induced by ubiquitous stimuli not only cytokines and growth factors but also heme (its substrate) and antioxidant. HO-1 and the heme catabolism play pleiotropic effects in preventing injury caused by many diseases [11–15]. Considerable studies showed that HO-1 possesses cytoprotective, proangiogenic, and anti-inflammatory effects in cardiovascular diseases [16–18]. Biliverdin can be transformed into bilirubin catalyzed by NADPH biliverdin reductase. Bilirubin and biliverdin are powerful antioxidants [19]. Carbon monoxide (CO), known as a gaseous signaling molecule, executes various biological functions such as reducing oxidative stress and regulating inflammation [20].

Hydrogen sulfide (H_2_S) is known for its odor and poison, but massive evidences have indicated that it possesses various important roles in physiology and pathophysiology. The same as nitric oxide (NO) and carbon monoxide (CO), hydrogen sulfide is regarded as a gaseous signaling molecule. Increasing studies indicate that hydrogen sulfide executes various biological functions such as reducing oxidative stress [21, 22] and regulating inflammation [23]. Our previous study suggests that hydrogen sulfide accelerates the wound healing in rats with diabetes [24].

In the present study, we aimed to evaluate the effects of increased expression of HO-1 protein on delayed wound healing via examining the change of inflammatory cytokines such as TNF-*α* and IL-6, antioxidant, and angiogenesis in diabetic rats.

## 2. Materials and Methods

### 2.1. Materials

Chloral hydrate, streptozotocin, hemin, and a horseradish peroxidase-conjugated secondary anti-rabbit antibody were purchased from Sigma (Sigma Chemical Co., St. Louis, MO, USA). Rabbit polyclonal antibodies *β*-actin, CYP-5 were purchased from Bio Basic Inc. (Canada), and HO-1, TNF-*α*, IL-6, CBS, and CSE were obtained from Wuhan Boster Bioengineering Limited Company (Wuhan, China).

### 2.2. Animals and Preparation of Diabetic Rats

Male Sprague-Dawley (SD) rats weighing 240–280 g were provided by the Animal Experimental Center in Wannan Medical College. All animal experiments were in compliance with Chinese Community guidelines for the use of experimental animals. Animals were raised in temperature-controlled animal laboratory with 22 ± 2°C and a twelve-hour alternate between light and dark. After 1 week, fasting rats were intraperitoneally injected with streptozotocin (STZ, freshly dissolved in 0.1 mol/L ice-cold sterile citric acid-sodium citrate buffer, pH 4.5) at dose of 65 mg/kg. Seventy-two hours after injection of STZ, fasting blood glucose was determined to confirm diabetes. Rats with blood glucose concentration >16.7 mmol/L were considered to have type 1 diabetes.

### 2.3. Preparation of Wounding in Rats

Two weeks after STZ treatment, the animals were randomly assigned to three groups: nondiabetic control rats (NDC); diabetic rats treated with vehicle (DTV); diabetic rats treated with hemin (DTH). Rats were housed individually with standard pellet diet and water ad libitum. Animals were anaesthetized with 10% chloral hydrate by intraperitoneal injection at a dose of 300 mg/kg. The hind dorsum was shaved and disinfected with 75% ethanol. A circular incision with full thickness was created on the dorsum skin from each rat. The wounds from NDC and DTV rats were treated with control cream, and rats from DTH group received 10% hemin ointment on wound. The sizes of the wounds were measured on days 5, 10, 15, and 20, respectively. Twenty-one days after ointment treatment, the rats were anaesthetized with chloral hydrate (300 mg/kg i.p.) and sacrificed, and fasting blood samples and granulation tissues from wound were collected for biochemical analyses.

### 2.4. Measurement of Wound Closure Rate

The wounds from each individual rat were digitally snapped. The wound closure in this experiment was quantified with Image-Pro Plus 5 software. The rate of wound closure was expressed as the ratio of wound closure: the wound closure rate (%) = (initial area − detected area)/initial area × 100%.

### 2.5. Measurement of Antioxidant

Fasting blood samples were centrifuged at 1300 ×g for separation of serum. Serum was used to determine MDA content and SOD activity by commercially available kits (Nanjing Jiancheng Bioengineering Institute, Nanjing, China).

### 2.6. Determination of VEGF and ICAM-1

Serum VEGF and ICAM-1 levels were determined with rat VEGF and ICAM-1 specific ELISA kit (Hefei Bomei Biotechnology Co., Ltd., China) according to kit instruction. The concentrations of VEGF and ICAM-1 in serum were expressed as ng/L and pg/mL, respectively.

### 2.7. Analyses of Coagulation

Prothrombin time, thrombin time, and fibrinogen were determined on semiautoanalyzer by the enzymatic colorimetric methods (STAGO, France) according to kit instruction.

### 2.8. Analyses of Histology

Wound samples were collected for histological study. Fixed wound tissues in 4% neutral formalin were embedded in paraffin and then sectioned at the thickness of five microns for Hematoxylin-Eosin (H-E) staining. Stained sections were used to evaluate reepithelialization, granulation tissue under a light microscope. The small blood vessels in wounds were counted in high power field.

### 2.9. Western Blotting

Wound tissues (0.2 g) were harvested and lysed in 2 mL of ice-cold lysis buffer with 2 mmol/L PMSF, 100 mmol/L Na_4_P_2_O_7_, 50 mmol/L HEPES, 10 mmol/L EDTA, 100 mmol/L sodium fluoride, 10 mmpl/L sodium orthovanadate, and protease inhibitor mixture for 15 min. Lysate was centrifuged at 13,000 g for 20 min at 4°C. Proteins denatured in supernatants were electrophoretically separated on an 8% stacking gel and following 12% sodium dodecyl sulfate-polyacrylamide gel and then transferred to 0.2-*μ*m nitrocellulose membranes. The membranes were blocked in TBS-T (10 mmol/L Tris, 150 mmol/L NaCl, and 0.1% Tween 20, pH 7.5) with 5% nonfat milk for 2 hours at room temperature and incubated with rabbit polyclonal antibodies *β*-actin (1 : 1000), CYP-5 (1 : 1000) (Bio Basic Inc., Canada), HO-1 (1 : 300), TNF-*α* (1 : 400), IL-6 (1 : 400), CBS (1 : 400), and CSE (1 : 400) (Wuhan Boster Bioengineering Limited Company, China) in TBS-T with 5% nonfat milk overnight at 4°C. After the membranes were extensively washed, a horseradish peroxidase-conjugated secondary anti-rabbit antibody (1 : 10 000 dilution; Sigma Chemical Co., St. Louis, MO, USA) was added and incubated for 2 hours. After being rinsed three times, the membranes were visualized by DAB (Bio Basic Inc., Canada).

### 2.10. Statistical Analysis

Data were presented as mean ± SE. Differences between the groups were analyzed with one-way analysis of variance (ANOVA) using least significance difference and *t*-test. Values of *P* < 0.05 were considered significant.

## 3. Results

### 3.1. General Characteristics

After injection of STZ, rats showed an increased concentration of fasting blood glucose and diabetic signs such as polyuria, increased water consumption, and weight loss.

### 3.2. Effects of Heme Oxygenase-1 on Wound Healing

**Figure 1:**
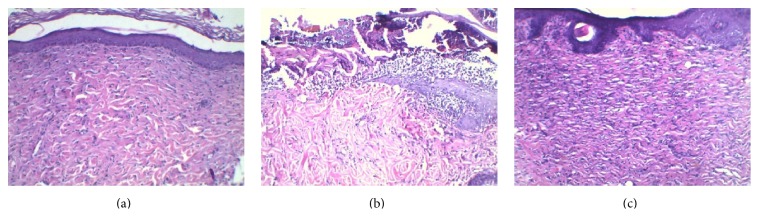
Photomicrographs of H-E staining in the wound tissue of each group (×100). (a) Nondiabetic control rats showed reepithelialization around the wound. (b) Inflammatory cell infiltration was observed in the wound from diabetic rats treated with vehicle. (c) Reepithelialization was observed in the wound from diabetic rats treated with hemin.

The wound closure was observed by measuring the change of the wound area and checking macroscopic differences of the wounds. The wounds were swelling and purulent and the closure rate of the wound was not significantly different in the next two days. Compared with DTV rats, heme oxygenase-1 significantly accelerated the wound closure rates from DTH rats after 5 days (*P* < 0.01) (Table 1). Treatment of hemin, an inductor of heme oxygenase-1, promoted the wound reepithelialization in diabetic rats (Figure 1). At the end of the experiment, the wounds from DTH rats were almost healed.

### 3.3. Effects of Heme Oxygenase-1 on Coagulation Activity

The change of coagulation activity is one of the factors on microcirculation, which play an important role in wound healing. The results from the study indicated that sustained hyperglycemia significantly shortened prothrombin time (PT) and thrombin time (TT) and increased level of fibrinogen in serum (*P* < 0.01) (Table 2). Compared with DTV rats, hemin treatment significantly extended PT and TT and reduced the level of fibrinogen in serum (*P* < 0.01) (Table 2).

### 3.4. Change of Antioxidant Effects

**Figure 2:**
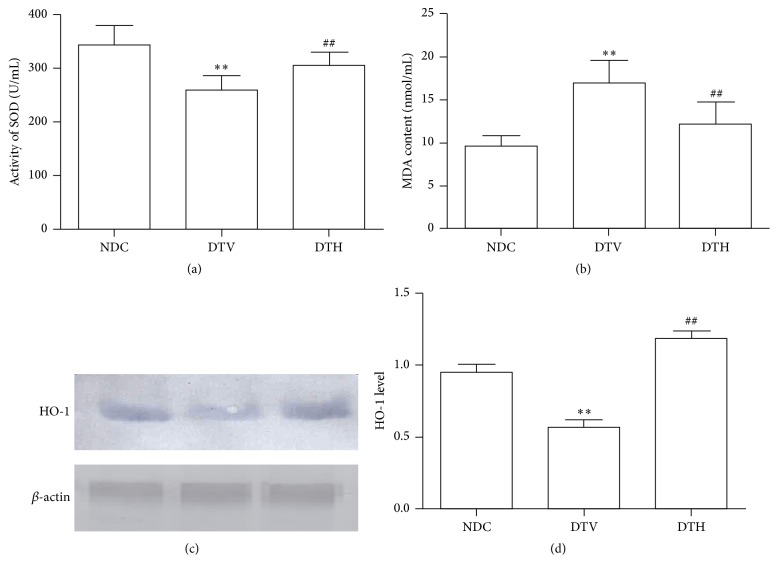
HO-1 enhanced antioxidant effects in diabetic rats. (a) Activity of SOD in serum was significantly corrected by induction of HO-1 in diabetic rats (^*∗∗*^ *P* < 0.01 versus NDC; ^##^ *P* < 0.01 versus DTV). (b) Induction of HO-1 reduced MDA content in serum from diabetic rats (^*∗∗*^ *P* < 0.01 versus NDC; ^##^ *P* < 0.01 versus DTV). (c) Expressions of HO-1 were determined. (d) Relative level of HO-1 was increased in diabetic rats treated with hemin (^*∗∗*^ *P* < 0.01 versus NDC; ^##^ *P* < 0.01 versus DTV).

HO-1 protein expression was reduced in wound tissue from diabetic rats when compared with NDC rats. Hemin treatment significantly induced HO-1 protein expression. Decreased activity of SOD and increased content of MDA were observed in diabetic rats (*P* < 0.01) (Figure 2). The inductor of HO-1 elevated the activity of SOD and lessened MDA content (*P* < 0.01) (Figure 2) in diabetic rats.

### 3.5. Effects of Heme Oxygenase-1 on the Inflammatory Responses

**Figure 3:**
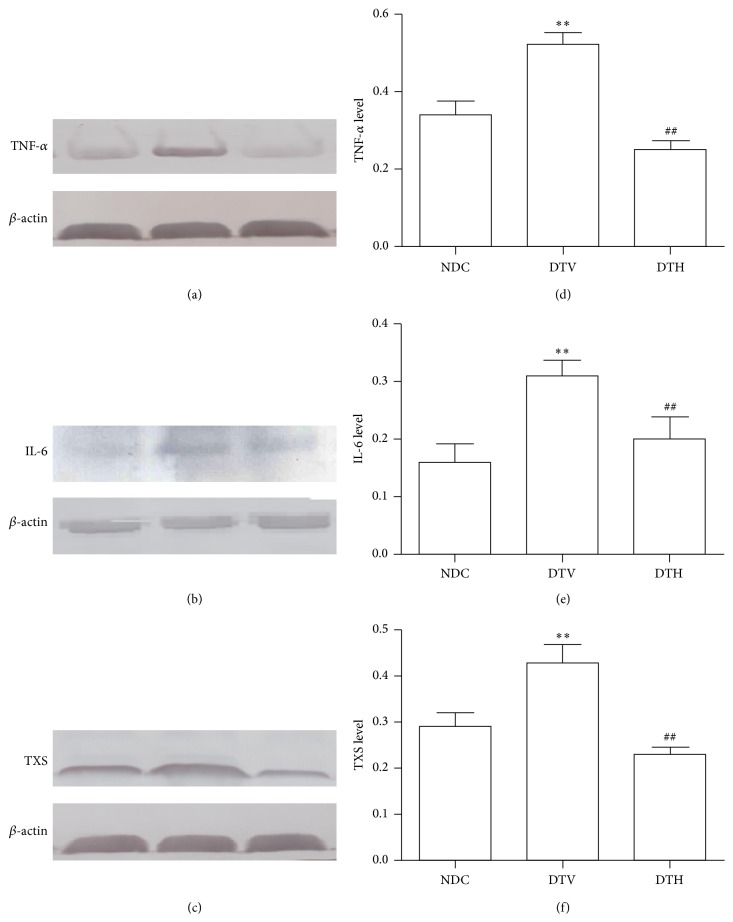
Induction of HO-1 elevated anti-inflammation in diabetic rats. HO-1 decreased expressions and relative levels of TNF-*α* ((a) and (d)), IL-6 ((b) and (e)), and TXS ((c) and (f)) (^*∗∗*^ *P* < 0.01 versus NDC; ^##^ *P* < 0.01 versus DTV).

The levels of TNF-*α* and IL-6 protein expression were significantly increased in the granulation tissues from DTV rats when compared with NDC rats (*P* < 0.01) (Figure 3). Inductor of HO-1 corrected the change. The results from the present study indicated the increase in the number of leucocytes and leukomonocytes (*P* < 0.05) (10^9^/L, 4.08 ± 0.56 versus 23.41 ± 2.25; 2.94 ± 0.63 versus 13.36 ± 2.76), and leukocyte infiltration was present in the wounds from DTV rats. Increase in the number of leucocytes and leukomonocytes was reduced in DTH rats (*P* < 0.05) (10^9^/L, 23.41 ± 2.25 versus 12.39 ± 0.94; 13.36 ± 2.76 versus 7.51 ± 0.74); there was almost no infiltration of leukocyte under the microscope in granulation tissues from DTH rats.

Thromboxane synthase (TXS) is an enzyme which catalyzes prostaglandin endoperoxide into thromboxanes. Our result indicated that expression of TXS was increased in wound tissues from diabetic rats when compared with control rats (*P* < 0.05) (Figure 3), and expression of TXS was reduced in granulation from DTH rats compared with DTV (*P* < 0.05) (Figure 3).

### 3.6. Effects of Heme Oxygenase-1 on Angiogenesis

**Figure 4:**
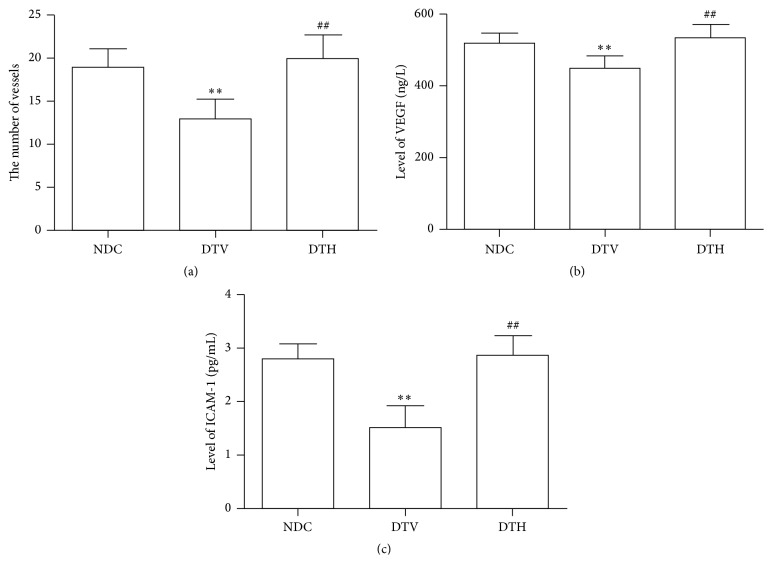
HO-1 ameliorated angiogenesis in granulation tissues from diabetic wounds. (a) The number of vessels was increased in granulation tissues from diabetic wounds treated with hemin (^*∗∗*^ *P* < 0.01 versus NDC; ^##^ *P* < 0.01 versus DTV). ((b) and (c)) HO-1 increased levels of VEGF and ICAM-1 in serum from diabetic rats (^*∗∗*^ *P* < 0.01 versus NDC; ^##^ *P* < 0.01 versus DTV).

To estimate microcirculation, we checked the number of the small vessels in the wound under microscope. The results showed that fewer vessels were observed in the granulation tissues from DTV rats compared with NDC rats (*P* < 0.05) (Figure 4). The number of vessels was near to normal in DTH rats. Furthermore, decreased levels of VEGF and ICAM-1, cytokines which affect angiogenesis, were observed in DTV rats when compared with NDC rats (*P* < 0.05) (Figure 4). Reduced levels of VEGF and ICAM-1 were improved by treatment with hemin (*P* < 0.05) (Figure 4).

### 3.7. Effects of Heme Oxygenase-1 on CSE and CBS Protein Expression

**Figure 5:**
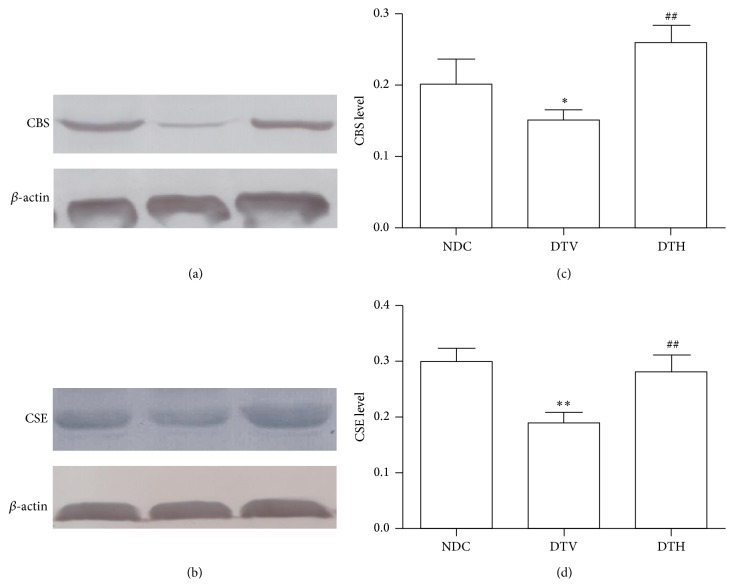
HO-1 improved the expression of hydrogen sulfide synthase. HO-1 increased expressions and relative levels of CBS ((a) and (c)) and CSE ((b) and (d)) (^*∗*^ *P* < 0.05 versus NDC; ^*∗∗*^ *P* < 0.01 versus NDC; ^##^ *P* < 0.01 versus DTV).

To explore the beneficial effect of heme oxygenase-1 on wound healing in diabetic rats and its possible mechanism, CSE and CBS protein expressions were determined. The results revealed that diabetes reduced the expressions of CSE and CBS protein (*P* < 0.05) (Figure 5), while the inductor of HO-1 restored diabetes-induced loss of CSE and CBS protein expression (*P* < 0.05) (Figure 5).

## 4. Discussion

In the present study, we evaluated the effect of induction of HO-1 on wound healing in diabetic rats. Our results showed that hyperglycemia impaired cutaneous wound closure from diabetic rats. Induction of HO-1 accelerated delayed wound closure by reducing inflammatory cytokines such as TNF-*α*, IL-6, increasing antioxidant, and improving angiogenesis in wound tissues from diabetic rats. Furthermore, inductor of HO-1 increased expression of CBS and CSE protein.

Poor wound healing represents one of the most serious diabetic complications. Sustained hyperglycemia impairs endothelial cells and leads to vascular dysfunction in wounds. Ischemia results in a serious infection and impairs formation of granulation tissue in diabetic wounds [25]. Therefore, the therapeutic angiogenesis is beneficial to wound healing in diabetes. Previous study verified that engraftment of bone marrow progenitor cells (BM-PCs) can stimulate angiogenesis and cell proliferation and promote wound closure in diabetic mice [26]. Excisional wound models have become effective methods of preclinical testing in diabetic animals [27].

Considerable evidence from both animal models and clinic indicates that oxide stress plays an important role in the occurrence and development of disease. Oxide stress results from excessive ROS and reduced antioxidant, which impairs vascular function [28, 29]. Sustained hyperglycemia can result in an increase of ROS production by various mechanisms, which aggravates oxide stress. Therefore, various tissues are easier to be damaged by ROS, and wound closure is impaired by microcirculatory disturbance resulting from injury of vessels in diabetes. Some studies showed that antioxidants and radical scavenger can improve diabetic complications and various diseases related to impairment of ROS [30, 31]. HO-1, called HSP 32, is thought to be an antioxidant enzyme [32]. HO-1 has protective effects on cells and various tissues by its antioxidant [29]. Our previous study suggested that increased expression of HO-1 can improve diabetic complications [30]. In the study, our results showed that HO-1 protein expression was reduced in wound tissue, while MDA, a product of lipid peroxidation, was significantly increased, and the activity of SOD was decreased in serum from diabetic rats. Furthermore, wound closure rate was declined. HO-1 was induced by hemin in the present study, and results showed that induction of HO-1 accelerated wound closure from diabetic rats. These suggested that enhanced antioxidant in wound from diabetic rats was in favor of wound closure.

Wound healing is a complex pathophysiological process involved with physiological events such as inflammation, angiogenesis, and reepithelialization. However, wound healing in diabetes was impaired by many factors including infection, hypoxia, and excess inflammation. Delayed wound healing is associated with increased inflammatory cytokines in diabetes [33–35]. Chronic inflammatory response increases wound infection in diabetic patients, which results in failure of diabetic wound to heal [36]. Previous studies showed that antioxidant treatment improves the delayed healing in diabetic wound via decreasing inflammatory response [37]. It has been showed that HO-1 is beneficial to cut down inflammatory process through reducing ROS production [38]. To examine the effect of HO-1 on the inflammatory response, we investigated the changes in expression of cytokines related with inflammatory response including TNF-*α* and IL-6. Our results suggested that elevated level of HO-1 downregulated the expressions of TNF-*α* and IL-6. Thromboxane synthase (TXS) is a hemoprotein with feature of the cytochrome P-450 family which catalyzes prostaglandin endoperoxide into thromboxanes [39]. Thromboxane production is increased in various inflammatory and cardiovascular diseases [40, 41]. It has been shown that thromboxane can activate and aggregate platelet and regulate vasoconstriction. Therefore, thromboxane exerts a vital effect in cardiovascular diseases such as coronary artery syndrome, atherosclerosis, and vessel remodeling [42–44]. Some thromboxane synthase inhibitors have been used in treatment of cardiovascular diseases through their anti-inflammation [45, 46]. In the present study, our results showed that TXS protein expression was increased in wound tissues from diabetic rats. While wounds were treated with hemin in diabetic rats, expression of TXS protein was reduced in wound tissue. The findings suggested that induction of HO-1 promoted wound healing via its anti-inflammation.

Blood vessels were impaired by ROS and inflammatory cytokine in diabetes, which led to disorder of coagulation and anticoagulation, and peripheral circulation failure [9, 10]. Ischemia and hypoxia resulting from circulation failure delayed the wound healing in diabetes. Therefore, improvement of circulation failure by angiogenesis and homeostasis between coagulation and anticoagulation may play an important role in diabetic wound healing. Our results indicated that the number of capillaries was increased in granulation tissue from diabetic wound treated with inductor of HO-1, which suggested that induction of HO-1 can promote angiogenesis. Furthermore, an increase in level of VEGF was found in diabetic rats treated with inductor of HO-1 when compared with diabetic rats treated with vehicle. VEGF is one of the key cytokines which increase vascular permeability, mediate migration and proliferation of endothelial cells, and thus stimulate angiogenesis [47–49]. In addition, level of ICAM-1 was increased in diabetic rats treated with hemin when compared with diabetic rats treated with vehicle. ICAM-1 is an inducible transmembrane protein, which can improve angiogenesis through regulating leukocyte adhesion to endothelial cells and activation of endothelial cells [50, 51]. The findings indicated that induction of HO-1 can accelerate wound closure via angiogenesis in diabetic wound.

Hydrogen sulfide (H_2_S) has been known as a physiologically and pathophysiologically active gasotransmitter [52, 53]. Endogenous hydrogen sulfide is generated by catalysis of cystathionine beta-synthase (CBS) and cystathionine *γ*-lyase (CSE) [54]. Increasing evidence indicates that hydrogen sulfide can exert antihypertensive effect through regulating vasorelaxation and hemodynamics [55, 56] and effect on diabetes and its complications, cardiovascular disease by its antioxidant and anti-inflammation [21, 23]. Furthermore, some studies show that hydrogen sulfide can regulate proliferation and migration of endothelial cells to improve angiogenesis [57, 58]. Reduced expressions of CBS and CSE have been found in diabetes [59]. In the present study, we demonstrated that expressions of CBS and CSE were decreased in wound tissues from diabetic rats, and induction of HO-1 increased the expressions of CBS and CSE.

In conclusion, our results reveal the beneficial effects of HO-1 on diabetic wound healing. HO-1 showed its anti-inflammatory response and antioxidant activity. Furthermore, the protective effects of HO-1 may be associated with its angiogenesis in granulation tissues from diabetic wounds. In addition, production of hydrogen sulfide may be involved in the effect of HO-1 on the wound healing in diabetes. Our results suggest that it is significant to further study the precise mechanism of HO-1 to heal diabetic wound for clinical application.

## Figures and Tables

**Table 1:** Effects of HO-1 on closure rate of wound.

	NDC	DTV	DTH
Day 5	23.0 ± 3.3	13.3 ± 3.3^*∗∗*^	20.6 ± 2.8^##^
Day 10	54.3 ± 4.0	33.2 ± 5.8^*∗∗*^	56.1 ± 6.6^##^
Day 15	81.7 ± 3.8	50.6 ± 8.0^*∗∗*^	84.6 ± 4.1^##^
Day 20	95.9 ± 3.1	74.8 ± 7.6^*∗∗*^	96.3 ± 3.9^##^

^*∗∗*^ *P* < 0.01 versus NDC; ^##^ *P* < 0.01 versus DTV.

**Table 2:** Effects of HO-1 on coagulation activity.

	NDC	DTV	DTH
PT (s)	12.3 ± 0.7	10.1 ± 0.6^*∗∗*^	11.1 ± 0.5^##^
TT (s)	29.4 ± 2.1	20.8 ± 2.7^*∗∗*^	27.1 ± 2.1^##^
Fibrinogen (mg/mL)	1.85 ± 0.16	2.96 ± 0.25^*∗∗*^	1.19 ± 0.18^##^

^*∗∗*^ *P* < 0.01 versus NDC; ^##^ *P* < 0.01 versus DTV.
